# Recent Progress in Detecting Enantiomers in Food

**DOI:** 10.3390/molecules29051106

**Published:** 2024-03-01

**Authors:** Changlong Hao

**Affiliations:** School of Food Science and Technology, State Key Laboratory of Food Science and Resources, Jiangnan University, Wuxi 214122, China; hcl@jiangnan.edu.cn

**Keywords:** food safety, amino acids, chiral pesticides, electrochemical method, fluorescent method

## Abstract

The analysis of enantiomers in food has significant implications for food safety and human health. Conventional analytical methods employed for enantiomer analysis, such as gas chromatography and high-performance liquid chromatography, are characterized by their labor-intensive nature and lengthy analysis times. This review focuses on the development of rapid and reliable biosensors for the analysis of enantiomers in food. Electrochemical and optical biosensors are highlighted, along with their fabrication methods and materials. The determination of enantiomers in food can authenticate products and ensure their safety. Amino acids and chiral pesticides are specifically discussed as important chiral substances found in food. The use of sensors replaces expensive reagents, offers real-time analysis capabilities, and provides a low-cost screening method for enantiomers. This review contributes to the advancement of sensor-based methods in the field of food analysis and promotes food authenticity and safety.

## 1. Introduction

Chirality is a ubiquitous property in nature and can be found throughout the universe. The non-superimposable stereoisomers are known as enantiomers, and a mixture of equal amounts of enantiomers is referred to as a racemate. Chiral molecules are present in various biological entities, including sugars, proteins, and nucleic acids, and they play essential roles in vital biological processes.

Food products contain a broad spectrum of chiral substances, such as sugars, amino acids, proteins, chiral additives, and flavor compounds [1]. Interestingly, chirality is also important to the perception of different tastes by humans. For example, the distinctive aroma of passion fruit and wine may be attributed to the presence of enantiomeric isomers in their flavor compounds. In the 1970s, scientists reported enantiomers with different odors, such as citronellol, linalool, and carvone. The detection of chiral substances in food holds significant importance as it can authenticate food products and ensure their safety. Analyzing the enantiomeric forms of amino acids serves as an example where an elevated concentration of d-alanine in milk can reveal microbial contamination. Additionally, the determination of the d-proline content in wine and vinegar can be utilized to ascertain the fermentation year of the product.

Amino acids are commonly found in food, such as apples, tomatoes, and goat milk, and they contain significant amounts of d-Ala, d-Ser, etc. Different processing methods can convert l-amino acids to their d-enantiomers, thereby affecting the taste and nutritional composition of food. Within the healthcare sector, deviations from normal levels of d-amino acids have been linked to a range of diseases and medical conditions. For instance, Alzheimer’s disease patients exhibit abnormal levels of d-Asp and d-Ser in their brain tissues, while cataract patients show abnormal levels of d-Glu and d-Asp in the eye lens. Elevated levels of d-Ala are often detected in gastric fluid from gastric cancer patients. Therefore, it is crucial to establish reliable and rapid methods for identifying amino acid enantiomers to investigate protein transformation and synthesis in the human body, guide food production, and provide diagnostic criteria for various diseases.

Another common type of chiral substance found in food is chiral pesticides [2,3,4,5,6]. Chiral pesticides exist as one or more enantiomers with different spatial configurations, resulting in significant differences in their pharmacological effects, degradation rates, and toxicity levels [7,8,9,10,11,12,13,14]. Analyzing the residual presence of chiral pesticides in food is necessary to maximize their efficacy while minimizing their adverse effects on non-target organisms. Current pesticide residue analysis in food primarily focuses on the total amount of a specific pesticide residue in accordance with regulatory requirements. However, there is a growing need to determine the enantiomeric composition of chiral pesticides in food to provide targeted detection methods and meet the demands of regulatory standards.

Various analytical techniques have been employed to determine enantiomers in food [15,16,17,18,19,20,21,22], including liquid chromatography, spectrophotometry, capillary electrophoresis, and fluorescence analysis. While techniques like high-performance liquid chromatography can effectively identify and separate enantiomers, they suffer from time-consuming procedures, expensive instrumentation, and limited real-time analysis capabilities. Therefore, the imperative pursuit lies in the advancement of sensor-based methodologies that enable swift and real-time analysis of enantiomers in food samples. However, traditional chromatographic methods still provide a solid foundation for sensor-based approaches. For instance, cyclodextrins, crown ethers, and polysaccharide derivatives, which serve as chiral selectors, continue to play significant roles in sensor-based methods. Utilizing sensors for enantiomer identification not only replaces expensive reagents but can also be applied to industrial-scale production and serve as a cost-effective and expeditious screening approach; it provides a means to rapidly detect and analyze enantiomers.

In this review, we will delve into the latest progress made in the field of biosensors, examining the recent breakthroughs and advancements for enantiomer detection in food [23,24,25,26,27,28,29,30,31,32,33,34,35,36,37,38,39,40,41,42,43,44,45,46,47,48,49,50]. We will discuss the principles underlying these biosensors and highlight their applications in food analysis. Moreover, we will present the most significant developments in analytical techniques employed for chiral compound analysis in food matrices, focusing on publications from the past five years. By examining these advancements, this review aims to address the need for rapid and accurate analysis of enantiomers in food using biosensor technologies.

## 2. Detection Methods for Amino Acids

Amino acids, as the fundamental constituents of proteins, play a vital role in biological functions. Detecting the levels of amino acids in food can assess the quality and nutritional value of proteins. Different types and concentrations of amino acids can influence the biological utilization and absorption of proteins in the human body. The precise analysis and sensitive detection of amino acids play a vital role not only in deepening our understanding of the mechanisms by which amino acids function in living organisms but also in various fields such as the food industry, natural products, and medicine [23,24,25]. The content and ratio of d-amino acids can reflect the quality and freshness of food products. Thus, the development of efficient approaches to detect amino acids plays a crucial role in biological and food analysis.

Traditional methods face significant challenges in the structural identification and chiral recognition of amino acids. Based on different detection methods, amino acid detection can be mainly categorized into electrochemical and fluorescence detection. Sensors that enable the analysis of amino acid enantiomers with low detection limits generally rely on the utilization of highly sensitive sensing elements. The recognition unit for amino acid enantiomers plays a key role in sensor development, as the sensor’s recognition capability depends on the selectivity of the recognition unit. Developing a sensor for amino acid enantiomers requires the design of recognition sites that interact with different enantiomers. Since amino acid enantiomers possess chirality, the molecular structures used for recognition sites also exhibit chirality. Therefore, naturally occurring chiral molecules have received extensive attention, while novel materials with controllable structures have injected new vitality into the field of amino acid enantiomer recognition. In addition to chiral molecular structures, chiral sensing interfaces and environments can also contribute to the recognition of amino acid enantiomers. The interaction between amino acid enantiomers and chiral selectors often results in transient complexes without enantiomeric properties. The interaction between amino acid enantiomers and chiral selectors is typically achieved through non-covalent bonds, and stronger interaction forces facilitate the recognition of amino acid enantiomers. Therefore, the key to constructing sensors for amino acid enantiomers lies in the selection of chiral materials and the construction of chiral sensing interfaces.

### 2.1. Electrochemical Methods for Amino Acid Detection

Electrochemical sensors offer advantages such as high reliability and sensitivity. They provide a simple recognition mode, straightforward recognition elements, and easy signal conversion at the molecular level [1,2]. The detection instruments are typically compact, with potential for miniaturization and portability. Generally, electrochemical sensors are based on three-electrode or two-electrode systems, with an electrochemical workstation and a potentiostat providing the detection platform. Sensors utilizing biological and nanomaterials as chiral selectors for amino acids have been widely reported. When amino acid enantiomers bind with different chiral recognition groups, they generate distinct electron transfers, resulting in different measurement signals. 

**Figure 1:**
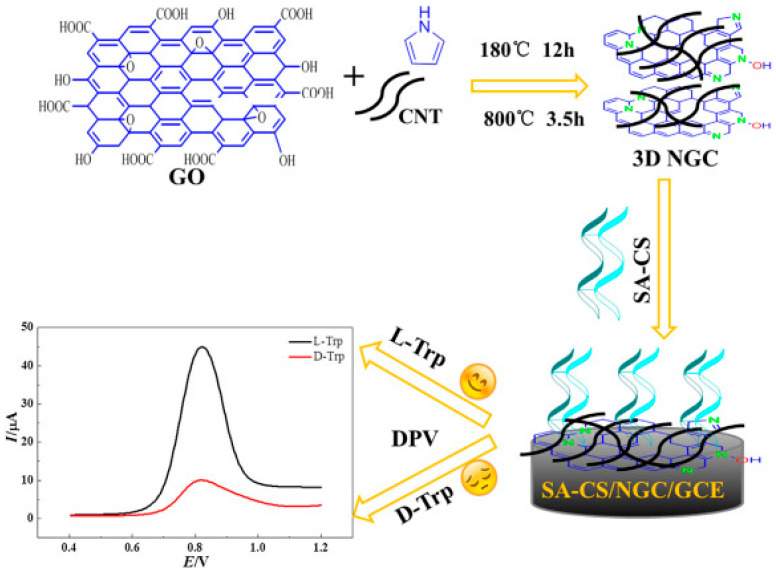
Schematic illustration of the fabrication of the SA-CS-NGC/GCE chiral sensing platform [3].

In 2020, Niu et al. developed a chiral interface by combining polysaccharides with N-doped graphene-CNT (NGC) as a substrate material [3]. This integration involved an amidation reaction between the carboxyl groups of sodium alginate (SA) and the amino groups of chitosan (CS), resulting in the formation of a chiral selector termed SA-CS. The chiral SA-CS-NGC interface can used for the electrochemical detection of tryptophan (Trp) enantiomers (Figure 1). Electrochemical measurements were conducted to evaluate the performance of the integrated polysaccharides/3D NGC interface in comparison to individual SA-CS and NGC components. The findings revealed that the integrated interface exhibited higher enantioselectivity toward l-Trp compared to d-Trp. 

The improved enantioselectivity can be ascribed to the reduced steric hindrance encountered by d-Trp during the formation of three-point interactions with the diastereoisomeric enantiomer-selector complexes. This allows l-Trp to detach more readily from the electrode modification layer and approach the electrode surface, thereby facilitating its detection. Furthermore, the preferential binding of SA-CS to l-Trp was validated when the chiral interface was used in real samples. This research contributes to the understanding of chiral discrimination mechanisms and provides insights into the design and development of chiral interfaces for electrochemical sensing applications. 

**Figure 2:**
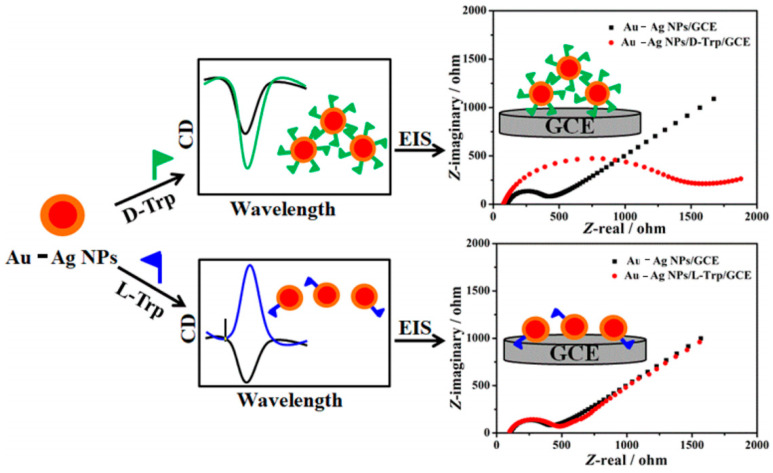
Schematic illustration of the potential mechanism of chiral discrimination using electrochemical impedance (EI) analysis [4].

In 2021, Au-Ag core–shell nanoparticles (Au-Ag NPs) were synthesized and applied to the highly sensitive detection of tryptophan (Trp) isomers using electrochemical impedance (EI) measurements (Figure 2) [4]. Circular dichroism analysis reveals that the Au-Ag NPs induce rotary polarization consistent with d-Trp but opposite to l-Trp, indicating selective binding of the Au-Ag NPs with d-Trp through preferential interactions. Upon comparing the Au-Ag NPs with the composites of d-Trp and Au-Ag NPs (Au-Ag NPs/d-Trp), a notable observation was made: the charge transfer resistance of the composites exhibited a significant increase. On the other hand, the charge transfer resistance of Au-Ag NPs/l-Trp remains relatively unchanged, indicating the weak affinity of Au-Ag NPs toward l-Trp. This selective binding and altered charge transfer behavior enable the ultrasensitive EI enantiodiscrimination of Trp isomers. Moreover, this approach successfully enables the highly sensitive determination of d-Trp within a low concentration range from 0.1 nM to 10 μM. The utilization of Au-Ag NPs as a sensing platform demonstrates its potential for the selective and highly sensitive determination of chiral compounds.

**Figure 3:**
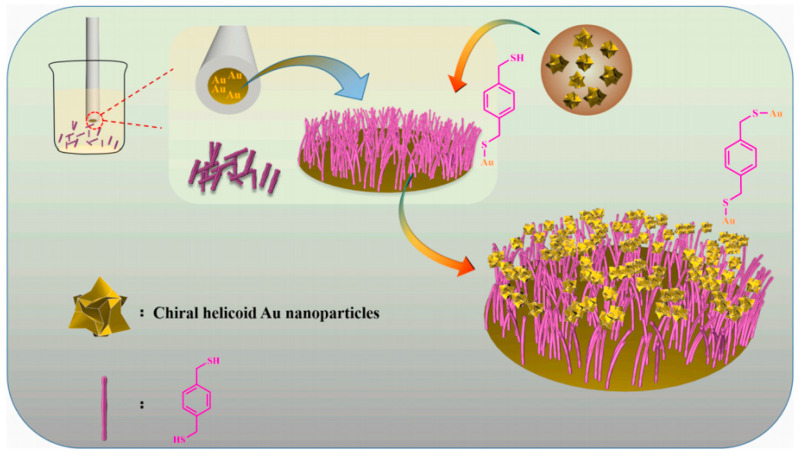
The fabrication process of chiral recognition interface for enantioselective electrochemical sensing [5].

In 2022, helicoid gold nanoparticles possessing inherent chirality were employed to develop an electrochemical chiral sensor, marking the first-ever utilization of such nanoparticles in this context (Figure 3) [5]. Significantly, the application of helicoid gold nanoparticles with inherent chirality in the construction of an electrochemical chiral sensor yielded an impressive nearly six-fold difference in peak currents for tyrosine (Tyr) enantiomers, as determined by the differential pulse voltammetry (DPV) method. This remarkable performance represents the most advanced achievement thus far in electrochemical enantiomer detection. Leveraging this substantial disparity in peak currents allows for precise determination of the enantiomeric purity and composition of Tyr analytes. Additionally, the developed chiral electrodes exhibited successful utilization in determining the l-Tyr content in diverse samples, encompassing capsules, beer, and milk. These findings underscore the immense potential of nanomaterials with intrinsic chirality as fundamental components for constructing chiral recognition interfaces that enable effective enantiomer discrimination. Moreover, these outcomes present promising prospects for the practical implementation of electrochemical chiral sensors.

A lot of electrochemical sensors have been employed to detect amino acid enantiomers [6,7,8,9,10,11,12,13,14,15,16]. The development of nanotechnology has brought significant advancements in materials science, medicine, and other fields. By modifying the surface of metal nanoparticles with chiral molecules, chiral nanomaterials have been widely employed for the recognition of amino acid enantiomers. The key to achieving effective results in this field lies in the design of amino acid enantiomer selectors. In addition to the enantiomer selectors mentioned earlier, commonly used selectors include chiral carbon quantum dots, chitosan, starch, and chiral polycyclic aromatic hydrocarbons.

### 2.2. Optical Sensing Methods

The enantiomer selector for amino acids is crucial in optical sensing devices. Optical sensors offer advantages such as ease of operation, rapid detection, high sensitivity, and low cost for detecting amino acid enantiomers. The structure of the chiral selector in this section is simpler compared to those used in electrochemical and quartz crystal microbalance methods. For instance, individual nanomaterials such as silver nanoparticles can be employed to discriminate amino acid enantiomers. This section will discuss the methods of detecting amino acid enantiomers using optical sensors.

#### 2.2.1. Surface Plasmon Resonance (SPR)

**Figure 4:**
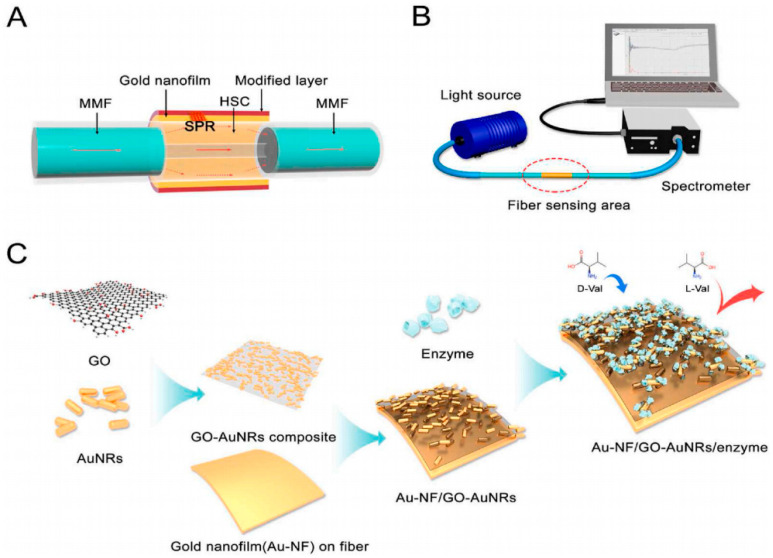
Schematic illustration of the all-fiber SPR d-AA sensor. (**A**) Longitudinal section of the fiber sensor area and (**B**) schematic illustration of the detection system. (**C**) Construction of a d-AA sensor assembled with the nanomaterials and the enzyme [17].

SPR is an optical method that enables real-time detection of interactions between amino acid enantiomers and chiral selectors. It involves illuminating a metal film to generate surface plasmon waves, which measure changes in metal surface curvature caused by the interaction between the target analyte and the sensing layer. SPR has extensive applications in the field of detecting amino acid enantiomers using biological materials. In 2020, Zhou et al. developed a chiral amino acid biosensor of the direct-assay type by integrating fiber optic SPR with an enzyme–substrate recognition mechanism [17]. An SPR sensor, as shown in Figure 4, was developed by immobilizing amino acid oxidase (ReDAAO) onto a composite material of graphene oxide and gold nanorods (GO-AuNRs). The constructed biosensor demonstrated the capability to differentiate amino acid isomers. Specifically, it demonstrated selective detection of d-amino acids within a linear concentration range spanning from 5 × 10^−4^ mM to 30 mM. Additionally, the biosensor exhibited excellent resistance to enantiomeric interference. The coupling of the enzyme ReDAAO, GO-AuNRs, and SPR technology provides a promising approach for chiral recognition. 

#### 2.2.2. Circular Dichroism Sensor for Amino Acid Detection

**Figure 5:**
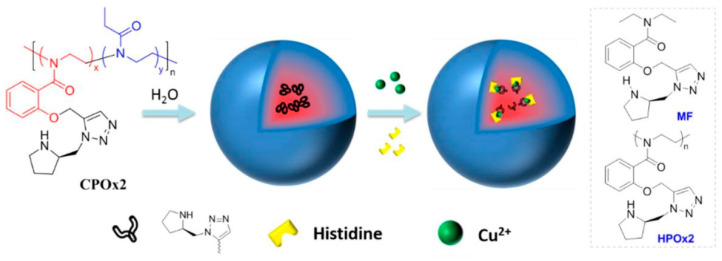
Scheme of the self-assembly of the amphiphilic copolymer CPOx2 and the sensing process, as well as the structure of monomeric (MF) and homopolymeric (HPOx2) analogs [18].

Circular dichroism (CD) spectroscopy is another commonly used optical method for sensing and recognizing amino acid enantiomers. Histidine is a crucial amino acid that the human body requires, and its concentration variation has been associated with liver and kidney diseases. In 2019, poly(2-oxazoline) derivatives with chiral pyrrolidine–triazole moieties in the side chain were used as chiral sensors for histidine (Figure 5) [18]. The findings of the study indicated that the homopolymer HPOx2 selectively binds optically active histidine through nitrogen/Cu^2+^ coordination, resulting in the formation of complexes that exhibit induced CD signals corresponding to the absolute configuration of the histidine molecule. Notably, the micelle-type nanoparticles assembled from the amphiphilic copolymer (CPOx2) display a significantly higher CD activity, with an intensity five times higher than that of the small-molecule or homopolymer counterparts. This innovative approach utilizing the chiroptical probe allows for the enantioselective analysis of histidine. The enhanced CD response observed in the micelle-type nanoparticles suggests their potential application in biomedical assays and chiral drug synthesis. Overall, this work presents a new approach to sensitively and selectively determine histidine and opens avenues for further research in the field of chiral sensing and biomedical applications.

#### 2.2.3. Fluorescence Sensor for Amino Acid Detection

Fluorescence sensors based on photo-induced luminescent amino acids offer several advantages, including high selectivity, real-time analysis, high sensitivity, and high throughput. Among various approaches in this field, the most widely applied method is the use of fluorescence sensors for the recognition and analysis of amino acid enantiomers. Numerous innovative nanomaterials have been utilized as sensing probes for the recognition of amino acid enantiomers. Among them, carbon quantum dots stand out due to their remarkable optical properties, high solubility in water, good biocompatibility, environmentally friendly nature, abundance of raw materials, cost-effectiveness, and favorable biocompatibility.

**Figure 6:**
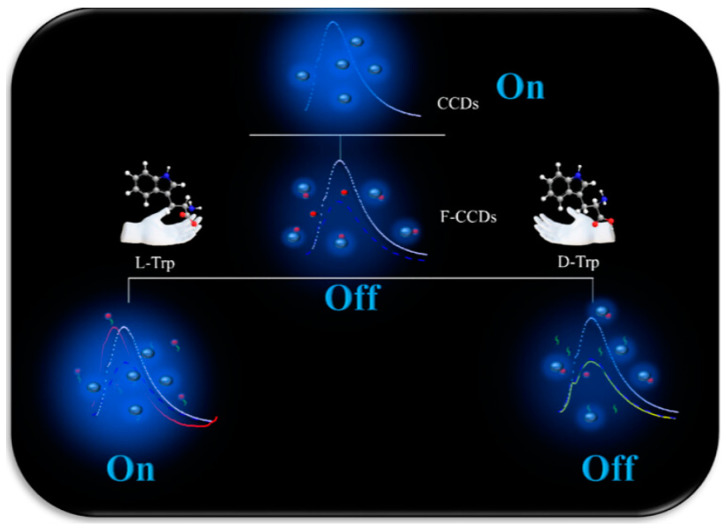
Scheme of chiral fluorescent carbon dots for tryptophan enantiomers identification [19].

In 2023, Li et al. employed chiral fluorescent carbon dots (CCDs) to successfully discriminate between the enantiomers of tryptophan (Trp) (Figure 6) [19]. The CCDs were synthesized via a hydrothermal reaction followed by chiral modification. Then, Fe^3+^ ions were complexed with CCDs to create a fluorescent probe, Fe^3+^-CCDs (F-CCDs). Notably, l-Trp exhibited a significant enhancement in the fluorescence of F-CCDs accompanied by a blue shift, while d-Trp had no impact on the fluorescence. F-CCDs demonstrated a low limit of detection (LOD) for l-Trp, with LOD values as low as 3.98 μM. 

In addition, other nanomaterials, such as carbazole-conjugated covalent organic frameworks and chiral assembly of ZnTPyP and CdTe quantum dots, can also serve as fluorescence probes for the recognition of amino acid enantiomers. Enzymes have been widely employed in biologically derived materials. For instance, a novel biosensor was developed by combining amino acid oxidase and peroxidase with zeolitic imidazolate framework-8 (ZIF-8), demonstrating excellent fluorescence recognition capabilities for various amino acid enantiomers [20]. Chiral Schiff bases, which are commonly used chiral organic structures, can also be employed in constructing fluorescence sensors for the discrimination of amino acid enantiomers. A novel ferrocene-based chiral Schiff base exhibited fluorescence sensing capabilities for various amino acid enantiomers, including methionine, alanine, serine, and histidine [21]. Collectively, these investigations emphasize the promise of employing biological materials as fluorescence probes in the field of amino acid enantiomer recognition. This expands the repertoire of available options and opens up new avenues for research in this area.

#### 2.2.4. SERS Method for Amino Acid Detection

**Figure 7:**
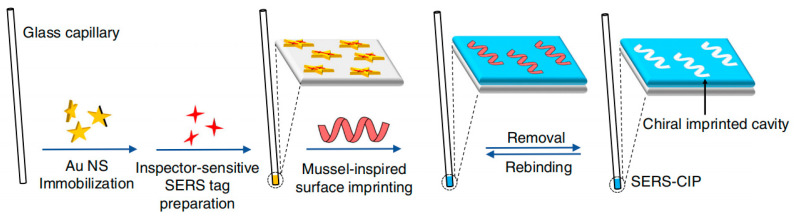
Schematic illustration of the SERS-CIP construction [22].

In 2022, Chen’s group developed a surface-enhanced Raman scattering-based chiral molecular imprinting strategy (SERS-CIP) for the highly selective and sensitive analysis of chiral amino acid enantiomers, including arginine, histidine, and aspartic acid, in seawater (Figure 7) [22]. Chiral molecularly imprinted polymers (CIP) possess cavities that are complementary to the target amino acid molecules in terms of shape, size, and functional groups. These cavities enable highly specific binding of the target chiral molecules, demonstrating unique advantages in chiral amino acid recognition. However, the involvement of inevitable nonspecific binding due to interactions between the polymer framework and the functional groups of chiral molecules has been a challenge. To address this issue, the research team developed an advanced CIP recognition mechanism and improved the specificity of CIP for enantiomer recognition by suppressing nonspecific binding. After conducting a detailed study on the sources of nonspecific binding in CIP using SERS, the team developed a detection and identification mechanism to explore the spatial state of CIP, thereby distinguishing between specific and nonspecific binding of chiral amino acid enantiomers. Through tests on enantiomeric selectivity, analysis of racemic mixtures, and chiral recognition in complex real samples, it was demonstrated that this mechanism meets the requirements of an ideal chiral recognition strategy and exhibits excellent practicality.

### 2.3. Quartz Crystal Microbalance (QCM) for Amino Acid Detection

**Figure 8:**
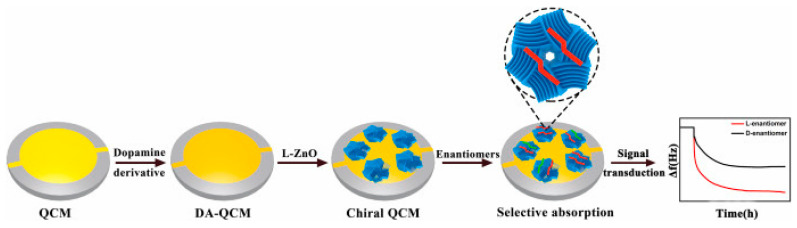
Schematic illustration of QCM sensor based on chiral ZnO for enantiomer discrimination [23].

QCM is a widely used label-free sensing platform capable of long-term detection of sub-nanogram-level changes. It operates by fabricating a disc using a piezoelectric material that is modified with a recognition layer. This setup enables the measurement of frequency changes resulting from the interaction between analytes and recognition molecules. When it comes to detecting amino acid enantiomers using QCM, the recognition of enantiomers by chiral selectors remains crucial. However, a significant challenge in utilizing QCM for amino acid enantiomer recognition lies in effectively immobilizing the chiral selectors onto the piezoelectric material. In 2022, chiral zinc oxide (ZnO) was used as a selector for constructing a QCM probe for enantioselective discrimination of amino acids (Figure 8) [23]. 

The chiral ZnO was synthesized using a self-assembly strategy induced by methionine, resulting in high topological chirality as confirmed by circular dichroism spectrum analysis. The sensor’s performance in chiral discrimination was evaluated by measuring frequency shifts in response to different amino acids, including aspartic acid, phenylalanine, lysine, and arginine, on the l-ZnO surface. The results showed that the sensor achieved chiral discrimination factors of 1.89 ± 0.04, 1.76 ± 0.11, 1.66 ± 0.07, and 1.54 ± 0.09 for aspartic acid, phenylalanine, lysine, and arginine, respectively. Significantly, the l-enantiomers demonstrated enhanced adsorption on the l-ZnO surface in comparison to their d-counterparts. In addition, molecular docking techniques were employed to uncover the recognition mechanism, revealing that chiral discrimination was influenced by distinct steric interactions between the enantiomers and the chiral ZnO surface.

### 2.4. Other Methods for Amino Acid Detection

**Figure 9:**
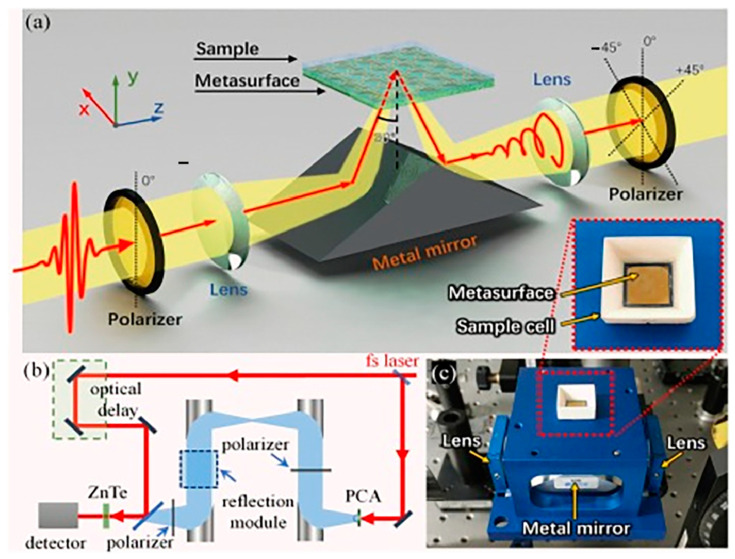
Experimental system: (**a**) an illustrative diagram depicting the experimental measurement principle and the key optical devices. (**b**) Light path of RTDPS. (**c**) Photograph of reflection module. The inset image is the photograph of the sample cell [24].

The utilization of terahertz (THz) sensing provides notable benefits for biochemical detection, including online, real-time, non-contact, and label-free analysis. Nevertheless, the detection of chiral solution samples using THz technology poses challenges. In 2021, Zhang et al. proposed a novel THz sensing method based on a THz reflection time-domain polarimetric spectroscopy (RTDPS) system and chiral metasurface sensor (Figure 9) [24].

This sensing method was used to identify the enantiomers present in three aqueous solutions of amino acids. Compared to conventional THz resonant sensing techniques, this strategy demonstrated substantial enhancements in sensitivity and detection accuracy for amino acid solutions. The achieved detection accuracy for various amino acid samples ranged from 10^−5^ to 10^−4^ g/mL, with a LOD of 1 × 10^−5^ g/mL. Furthermore, distinguishing d-and l-enantiomers with identical concentrations was made possible by the notable discrepancies in THz polarization parameters at specific frequencies. This approach proves versatile, extending beyond amino acid aqueous solutions to encompass other chiral biochemical solutions. Consequently, the combination of terahertz polarimetric spectroscopy and chiral surface sensors exhibits immense potential in achieving highly sensitive quantitative detection and precise chiral identification for biochemical material analysis.

**Figure 10:**
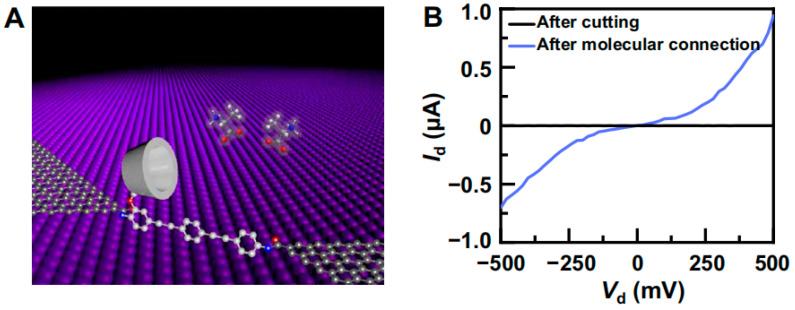
(**A**) Schematic diagram of permethylated-β-cyclodextrin (PM-β-CD)-based graphene–molecule–graphene single-molecule junctions (GMG-SMJs). (**B**) I–V curves of GMG-SMJs after oxygen plasma cutting and after further molecular connection [25].

In 2021, Guo’s group developed a single-molecule device platform for single-molecule electrical detection [25]. This platform enables the precise detection of the structure, chirality, and charge state of individual amino acid molecules at the level of a single molecule. The method relies on measuring the dynamic interactions between host and guest molecules using graphene–molecule–graphene single-molecule junctions (GMG-SMJs). These junctions are formed by sandwiching a molecular machine between nanogapped graphene point contacts through covalent bonding. The molecular machine incorporates a stable and conductive conjugated organic molecular framework, along with a recognition function facilitated by a permethylated-β-cyclodextrin (PM-β-CD) side arm. The developed system demonstrates accurate differentiation between various amino acids and their enantiomers (Figure 10). Using such devices, high-resolution electrical signals were recorded to achieve in situ real-time detection of the host–guest dynamics between amino acids and cyclodextrin molecules. The platform demonstrated the capability to detect four commonly occurring amino acids (alanine, tryptophan, serine, and tyrosine) and distinguish between their various forms. The binding process between amino acids and cyclodextrin is a second-order reaction, while the dissociation reaction is a first-order reaction. In aqueous solutions, amino acids exist in three different forms: zwitterions, conjugate acids, and conjugate bases. Each of these forms can engage in host–guest recognition with cyclodextrin. By conducting a thorough analysis of the dynamic states and employing theoretical calculations, it becomes possible to identify complex conductive states. Based on the parameters of amino acid dissociation relaxation time and the rate of molecular bridge conductivity change, a “fingerprint” of amino acid chiral recognition was generated. This allows for rapid and accurate identification of amino acid structure, type, and enantiomer. 

## 3. Detection of Chiral Pesticides

Modern agricultural production heavily relies on the use of pesticides to safeguard crop yields. However, the excessive and improper use of pesticides has led to increasingly severe food safety issues, posing a significant threat to human health. Thus, it is essential to strengthen the efficient detection of pesticide residues to ensure food quality and safety control. Currently, the national standard methods for pesticide residue detection include chromatographic methods and chromatography-mass spectrometry techniques. In China, around 40% of the pesticides employed are classified as chiral pesticides. These pesticides exhibit distinct variations in activity, toxicity, and environmental behavior between their enantiomers despite sharing similar physicochemical properties.

However, most chiral pesticides are still sold and used as racemic mixtures, raising concerns about the rationality of this form. The efficacy and safety of chiral pesticides cannot be solely assessed based on racemic evaluations but require careful consideration of the differences in activity, toxicity, and behavior between enantiomers. Although there are numerous sensors available, such as electrochemical biosensors, optical biosensors, surface-enhanced Raman spectroscopy techniques, polymerase chain reaction methods, and immunosensing technologies, for detecting pesticide residues in food, many of these methods fail to distinguish between enantiomers.

Currently, widely used methods for detecting chiral pesticide residues in food include liquid chromatography, gas chromatography, capillary electrophoresis, and capillary electrochromatography, which are also applied for the separation of chiral pesticides [26,27,28,29,30,31,32,33,34,35,36,37,38,39,40,41]. Supercritical fluid chromatography and ultra-high-performance liquid chromatography have also been studied for chiral pesticide separation. These methods offer advantages such as high sensitivity, accurate quantification, and high resolution. Nevertheless, these chiral pesticides face certain limitations that hinder their practical implementation in field applications. These limitations include the complexity of sample preparation, the high costs associated with their analysis, and the need for large and precise instruments.

Hence, there is a pressing need to develop sensing strategies that are efficient, convenient, sensitive, and rapid in detecting pesticide residues. Such advancements are of paramount importance as they play a crucial role in safeguarding food safety and protecting human health. In the following sections, we will provide an overview of several sensors, including electrochemical sensors and fluorescence sensors, that can be used for detecting chiral pesticides.

### 3.1. Sensors for Chiral Pesticide Detection Based on Electrical Signals

**Figure 11:**
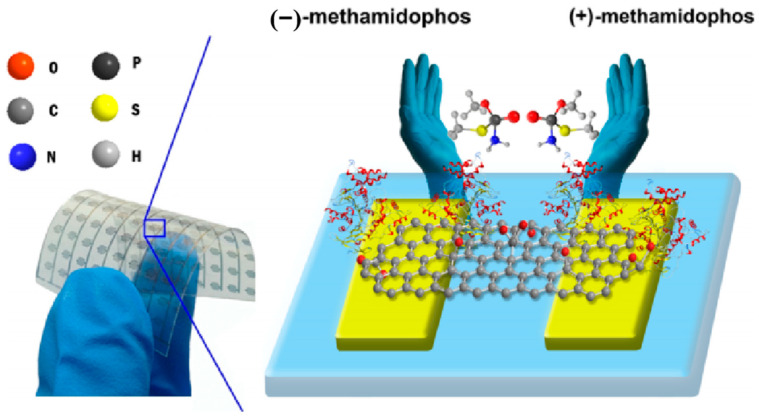
Scheme of one acetylcholinesterase-modified electrochemically reduced graphene oxide (AChE-ERGO) sensor and the chemical structure of the methamidophos enantiomers [42].

Graphene, an emerging material, has found extensive applications in the sensing field owing to its remarkable features, such as a large specific surface area, exceptional adsorption capacity, excellent conductivity, and favorable biocompatibility. In 2019, Zhang et al. achieved convenient, rapid, and real-time detection of the chiral pesticide malathion by combining acetylcholinesterase (AChE) and electrochemically reduced graphene oxide (ERGO) [42]. AChE, as the receptor protein for malathion in insects, exhibits strong sensitivity to malathion molecules, although their binding process falls within the realm of chemistry. Therefore, by combining ERGO with AChE, the toxicity difference between chiral pesticides and AChE can be converted into differences in electrical signals, enabling molecular detection at lower concentrations. The sensor was tested and demonstrated a detection limit of 0.32 μg/L and 0.34 μg/L for the enantiomers of malathion, effectively distinguishing between the two enantiomers even at a concentration of 10^−3^ mg/L (Figure 11) [42]. 

### 3.2. Electrochemical Sensors for Chiral Pesticide Detection 

Metal–organic framework (MOF) materials consist of porous crystalline structures composed of metal ions or clusters that are coordinated with organic ligands. MOF materials provide a versatile platform that finds applications in diverse fields, including gas storage, catalysis, and biosensing. MOF, in combination with synergistic recognition from molecularly imprinted membranes, can effectively address the issue of interference between enantiomers of chiral pesticides, providing new research insights and methods for the selective recognition and highly sensitive detection of chiral pesticides.

**Figure 12:**
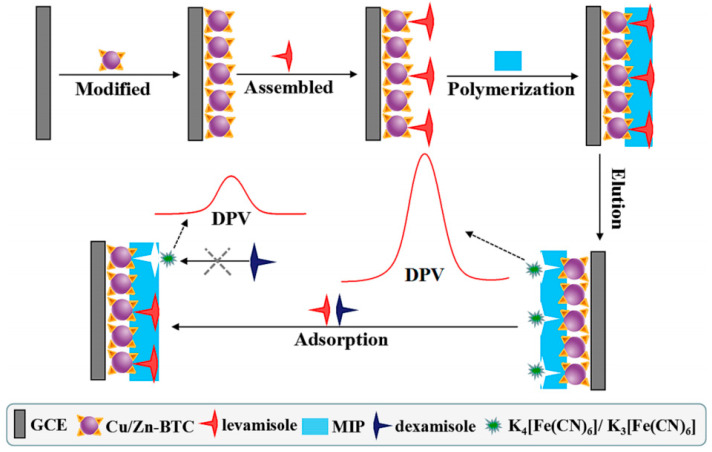
Schematic demonstrating the detection mechanism of levamisole through the utilization of an innovative electrochemical chiral sensor [43].

Li et al. devised a chiral electrochemical sensor that employed a synergistic enhancing recognition strategy for the sensitive detection of trace amounts of levamisole residues in food and the environment. This innovative approach successfully eliminated interference from dextro levamisole, enhancing the accuracy and reliability of the detection process (Figure 12) [43]. Firstly, to serve as the unit for molecular immobilization and signal amplification, a metal–organic framework material known as Cu/Zn-(benzene-1,3,5-tricarboxylic acid) (Cu/Zn-BTC) was synthesized. Subsequently, molecularly imprinted polymer (MIP) recognition units specific to levamisole were prepared on a Cu/Zn-BTC-modified glassy carbon electrode using levamisole as a template. After elution with an organic solvent, the sensor retained specific recognition sites for levamisole, enabling efficient recognition and binding, leading to a signal response. The sensor exhibited enhanced recognition capabilities for levamisole due to the incorporation of Cu/Zn-BTC and MIP as dual-recognition elements, effectively eliminating interference from the enantiomer dextro levamisole. The chiral probe was successfully used for the detection of levamisole in chicken meat and other real samples, achieving a LOD of 1.65 × 10^−12^ mol/L, surpassing existing national standard methods.

### 3.3. Fluorescence Sensors for Chiral Pesticide Detection

**Figure 13:**
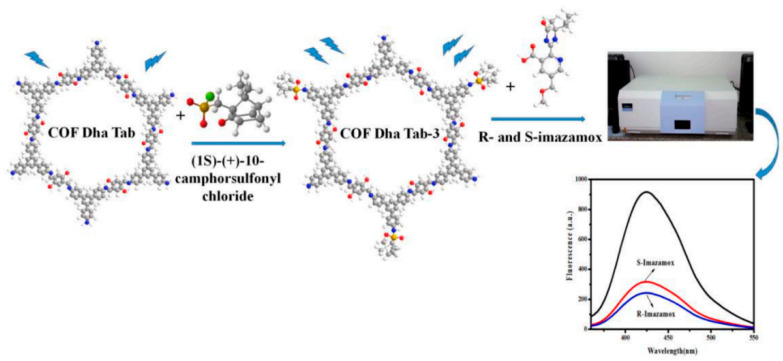
A fluorescent chiral covalent organic framework (COF) was synthesized through post-synthesis modification, enabling its application as an optosensor for the detection of imazamox enantiomers [44].

Imazamox is a herbicide belonging to the chemical class of imidazolinones. It is commonly used in agriculture to control a broad spectrum of grass and broadleaf weeds in various crops, including cereals, soybeans, and pulses. In 2023, Qin et al. reported the use of a covalent organic framework (COF) with intense chirality and fluorescence for imazamox enantiomer detection (Figure 13) [44]. The chiral COFs named Dha-Tab were synthesized via a series of chemical reactions. Firstly, 2,5-dihydroxyterephthalaldehyde (Dha) and 1,3,5-tris(4-aminophenyl)benzene (Tab) were reacted via a Schiff-base reaction. Then, a chiral modifier, (1S)-(+)-10-camphorsulfonyl chloride, was introduced through nucleophilic substitution. The resulting Dha-Tab COF exhibited impressive optical activity, robust covalent bond structure, high crystallinity, and a big specific surface area. Remarkably, Dha-Tab demonstrated specific enantioselectivity and rapid identification of imazamox enantiomers among five pesticide enantiomers, including S/R-imazamox, acephate, acetochlor, propisochlor, and metalaxyl. The LOD for S-imazamox and R-imazamox were 4.20 μmol/L and 3.03 μmol/L, respectively. Notably, the chiral COF exhibited a strong adsorption capacity for imazamox enantiomers, with a higher affinity for R-imazamox, as evidenced by the enantiomeric excess value of 5.30%. This chiral fluorescent COF represents a promising advancement in enantiomeric recognition capabilities.

Currently, the development of biosensors to determine chiral pesticide residues in food is still in the exploratory stage [45,46,47,48]. With the continuous advancement of nanotechnology, an increasing number of nanomaterials have emerged, providing support for the development of efficient, convenient, sensitive, and rapid sensing methods for pesticide residue determination. These innovative biosensors offer valuable enhancements to conventional methods and have the capability for real-time on-site measurement, making them highly promising in practical applications.

## 4. Detection of Other Chiral Substances in Food

In addition to amino acids and chiral pesticide residues, there are other chiral substances present in food, such as lactic acid, malic acid, and tartaric acid. Below is a brief introduction to the detection methods for these substances.

### 4.1. Detection of Malic Acid

Malic acid is a common food component, and measuring its content can be used for food quality control and nutritional assessment. By detecting the concentration of malic acid, the freshness, ripeness, and storage condition of food can be evaluated to ensure the quality meets standards and consumer expectations. In 2020, the specific chirality of single-walled carbon nanotubes was utilized as chiral selectors to construct biosensors for malic acid detection [49]. Different carbon-based substrates, such as graphite paste and graphite paste with carbon nanofiber-based paste, were used to investigate the sensor’s response to malic acid in potentiometric and differential pulse voltammetry (DPV) modes. When utilizing carbon nanofiber-based paste as the substrate, the multimodal sensor demonstrated chiral selectivity toward d-malic acid within the concentration range of 10^−3^ to 10^−15^ mol/L in the potentiometric mode and 10^−5^ to 10^−8^ mol/L in the DPV mode. On the other hand, the graphite paste-based sensor exhibited chiral selectivity toward l-malic acid within the concentration range of 10^−10^ to 10^−13^ mol/L in the potentiometric mode and 10^−4^ to 10^−7^ mol/L in the DPV mode. Interestingly, sensors employing graphene and chiral single-walled carbon nanotubes only exhibited chiral selectivity toward d-malic acid and responded exclusively in the DPV mode. These observations highlight the significant impact of the substrate on both the chiral selectivity and sensitivity of the measurements. This strategy was used for the detection of chiral malic acid in wine and apple juice samples. The method was found to be rapid, reliable, and capable of quantitatively determining l- and d-malic acid using different electrochemical principles after sample buffering. In real samples, the chiral analysis of malic acid demonstrated excellent recoveries (>90.00%) and low relative standard deviation (RSD) values (<1.00%).

### 4.2. Detection of Chiral Vapors in Food

**Figure 14:**
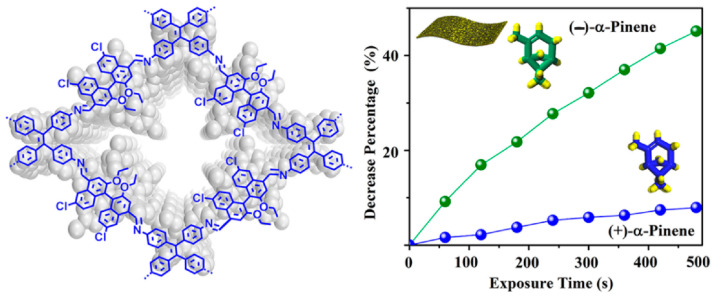
Schematic illustrating pinene detection by chiral BINOL-based covalent organic frameworks [50].

In 2019, Wu et al. reported the preparation and functionalization of COFs as chiral vapor sensors (Figure 14) [50]. The researchers utilized optically pure 1,1’-bi-2-naphthol (BINOL) as a chiral source to construct COFs. By carefully selecting enantiopure BINOL-based linear dialdehydes and aminophenyl derivatives as building blocks, two chiral fluorescent COFs with a 2D layered hexagonal or tetragonal structure were successfully synthesized. The COF incorporating flexible tetraphenylethylene units demonstrated the ability to be exfoliated into ultrathin 2D nanosheets and electrospun into free-standing nanofiber membranes. Both in solution and membrane systems, the fluorescence of the COF nanosheets was effectively quenched by chiral odor vapors through supramolecular interactions with the immobilized BINOL moieties. This resulted in highly sensitive and selective chiral vapor sensors.

Compared to BINOL-based homogeneous and membrane systems, the COF nanosheets demonstrated superior sensitivity and enantioselectivity. This enhancement was attributed to the confinement effect and conformational rigidity of the sensing BINOL groups within the COF framework. The successful incorporation of the beneficial BINOL chiral auxiliary into the open channels of COFs represents a great progress in the rational preparation of porous molecular materials for various applications.

## 5. Conclusions and Outlook

The analysis of enantiomers in food, such as amino acids, chiral pesticides, and chiral additives, holds significant importance. Consequently, the development of appropriate chiral sensing interfaces for the identification of chiral enantiomers in food has become a prominent area of research. This paper discusses various materials utilized for constructing sensing interfaces in different methods, focusing on the target analytes. These emerging biosensors take advantage of the unique properties of nanomaterials and offer several advantages. They provide enhanced sensitivity, selectivity, and stability, allowing for accurate and reliable analysis of enantiomers in food samples. Furthermore, by harnessing the potential of nanomaterials, it becomes possible to create compact and portable devices, facilitating quick and on-site analysis. The incorporation of nanotechnology into biosensors has revolutionized the detection of chiral enantiomers, introducing novel avenues for research and application. These groundbreaking biosensing strategies offer promising solutions to the challenges encountered in the analysis of pesticide residues, thereby holding the potential to transform the field of food safety. Future research in this area will further refine and optimize these biosensors, making them more practical and widely applicable in food industry settings.

The future direction in this field revolves around the advancement of electrochemical sensors for the precise detection of enantiomers in food. However, electrochemical sensors face interference from electroactive substances, which can affect the identification of enantiomers under certain electrochemical methods. In the future, the development of simple, low-cost, portable, and field-deployable sensors for quick analysis of chiral enantiomers in food remains a significant challenge. Chiral nanomaterials possessing distinct chiral configurations exhibit immense promise in the realm of chiral recognition within the food industry. Combining nanomaterials with traditional chiral selectors will provide new avenues for constructing chiral recognition sensing interfaces.

In conclusion, there is a need for further research and development to overcome the challenges associated with chiral enantiomer detection in food. By combining cutting-edge materials and state-of-the-art technologies, the field of chiral sensing in food will witness significant progress, leading to the establishment of dependable and feasible detection techniques for chiral enantiomers in food samples.

## Data Availability

Not applicable.

## References

[1] Niu X., Yan S., Zhao R., Han S., Cao K., Li H., Wang K. (2023). Chiral template–induced porphyrin-based self-assembled materials for electrochemical chiral sensing. Microchim. Acta.

[2] Niu X., Yang X., Li H., Liu J., Liu Z., Wang K. (2020). Application of chiral materials in electrochemical sensors. Microchim. Acta.

[3] Niu X., Yang X., Mo Z., Wang J., Pan Z., Liu Z., Shuai C., Liu G., Liu N., Guo R. (2020). Fabrication of an electrochemical chiral sensor via an integrated polysaccharides/3D nitrogen-doped graphene-CNT frame. Bioelectrochemistry.

[4] Gong L., Zhao Q., Wu S., Yin Z.-Z., Wu D., Cai W., Kong Y. (2021). Ultrasensitive Electrochemical Impedance Chiral Discrimination and Sensing of Tryptophan Isomers Based on Core–Shell-Structured Au–Ag Nanoparticles. Langmuir.

[5] Li F., Wu F., Luan X., Yuan Y., Zhang L., Xu G., Niu W. (2022). Highly enantioselective electrochemical sensing based on helicoid Au nanoparticles with intrinsic chirality. Sens. Actuators B Chem..

[6] Erbilen N., Zor E., Saf A.O., Akgemci E.G., Bingol H. (2019). An electrochemical chiral sensor based on electrochemically modified electrode for the enantioselective discrimination of D-/L-tryptophan. J. Solid State Electrochem..

[7] Yang X., Niu X., Mo Z., Wang J., Shuai C., Pan Z., Liu Z., Liu N., Guo R. (2019). 3D nitrogen and sulfur Co-doped graphene/integrated polysaccharides for electrochemical recognition tryptophan enantiomers. J. Electrochem. Soc..

[8] Wen T., Li J., Cai W., Yang B., Kong Y., Yin Z.-Z. (2023). A chiral sensing platform based on a multi-substituted ferrocene–cuprous ion complex for the discrimination of electroactive amino acid isomers. Analyst.

[9] Pei H., Wang J., Jin X., Zhang X., Liu W., Guo R., Liu N., Mo Z. (2022). An electrochemical chiral sensor based on glutamic acid functionalized graphene-gold nanocomposites for chiral recognition of tryptophan enantiomers. J. Electroanal. Chem..

[10] Moulaee K., Neri G. (2021). Electrochemical Amino Acid Sensing: A Review on Challenges and Achievements. Biosensors.

[11] Liu N., Liu J., Niu X., Wang J., Guo R., Mo Z. (2021). An electrochemical chiral sensor based on the synergy of chiral ionic liquid and 3D-NGMWCNT for tryptophan enantioselective recognition. Microchim. Acta.

[12] Jing P., Zhao C., Yin Z.-Z., Yang B., Li J., Cai W., Kong Y. (2022). An electrochemical chiral sensor based on competitive host–guest interaction for the discrimination of electroinactive amino acids. Analyst.

[13] Jing P., Yin Z.-Z., Cai W., Li J., Wu D., Kong Y. (2022). The hybrids of perylene tetracarboxylic acid functionalized multi-walled carbon nanotubes and chitosan for electrochemical chiral sensing of tryptophan enantiomers. Bioelectrochemistry.

[14] Imanzadeh H., Sefid-Sefidehkhan Y., Afshary H., Afruz A., Amiri M. (2023). Nanomaterial-based electrochemical sensors for detection of amino acids. J. Pharm. Biomed. Anal..

[15] Gao H., Lu Z., Xiao Y. (2022). An electrochemical chiral sensor for amino acids based on cyclodextrin modified thiophene-based copolymer. Carbohydr. Polym..

[16] Deng Y., Zhang Z., Pang Y., Zhou X., Wang Y., Zhang Y., Yuan Y. (2022). Common materials, extraordinary behavior: An ultrasensitive and enantioselective strategy for D-Tryptophan recognition based on electrochemical Au@ pL-cysteine chiral interface. Anal. Chim. Acta.

[17] Zhou Z., Yang Z., Xia L., Zhang H. (2022). Construction of an enzyme-based all-fiber SPR biosensor for detection of enantiomers. Biosens. Bioelectron..

[18] Liu K., Du G., Ye L., Jiang L. (2019). A chiroptical nanoprobe for highly selective recognition of histidine enantiomers in aqueous media. Sens. Actuators B Chem..

[19] Li J., Du N., Guan R., Zhao S. (2023). Construction of a Chiral Fluorescent Probe for Tryptophan Enantiomers/Ascorbic Acid Identification. ACS Appl. Mater. Interfaces.

[20] Liu G., Wang L., Zhu F., Liu Q., Feng Y., Zhao X., Chen M., Chen X. (2022). Facile construction of a reusable multi-enzyme cascade bioreactor for effective fluorescence discrimination and quantitation of amino acid enantiomers. Chem. Eng. J..

[21] Ucar A., Findik M., Bingol H., Guler E., Ozcan E. (2017). Organometallic chiral Schiff base for enantio-selective fluorescent recognition of methionine. Chem. Pap..

[22] Arabi M., Ostovan A., Wang Y., Mei R., Fu L., Li J., Wang X., Chen L. (2022). Chiral molecular imprinting-based SERS detection strategy for absolute enantiomeric discrimination. Nat. Commun..

[23] Du J., Xie F., Liu C., Ji B., Wei W., Wang M., Xia Z. (2023). Chiral zinc oxide functionalized quartz crystal microbalance sensor for enantioselective recognition of amino acids. Talanta.

[24] Zhang Z., Zhong C., Fan F., Liu G., Chang S. (2021). Terahertz polarization and chirality sensing for amino acid solution based on chiral metasurface sensor. Sens. Actuators B Chem..

[25] Liu Z., Li X., Masai H., Huang X., Tsuda S., Terao J., Yang J., Guo X. (2021). A single-molecule electrical approach for amino acid detection and chirality recognition. Sci. Adv..

[26] Zhu J., Yin L., Zhang W., Chen M., Feng D., Zhao Y., Zhu Y. (2021). Colorimetric Measurement of Deltamethrin Pesticide Using a Paper Sensor Based on Aggregation of Gold Nanoparticles. Coatings.

[27] An X., Pan X., Li R., Jiang D., Dong F., Zhu W., Xu J., Liu X., Wu X., Zheng Y. (2022). Enantioselective monitoring chiral fungicide mefentrifluconazole in tomato, cucumber, pepper and its pickled products by supercritical fluid chromatography tandem mass spectrometry. Food Chem..

[28] Guo P., An X., Chen W., Pan X., Li R., Xu J., Wu X., Zheng Y., Dong F. (2022). Separation and determination of fluindapyr enantiomers in cucumber and tomato and by supercritical fluid chromatography tandem mass spectrometry. Food Chem..

[29] Hergueta-Castillo M.E., López-Rodríguez E., López-Ruiz R., Romero-González R., Frenich A.G. (2022). Targeted and untargeted analysis of triazole fungicides and their metabolites in fruits and vegetables by UHPLC-orbitrap-MS2. Food Chem..

[30] Tao Y., Zheng Z., Yu Y., Xu J., Liu X., Wu X., Dong F., Zheng Y. (2018). Supercritical fluid chromatography–tandem mass spectrometry-assisted methodology for rapid enantiomeric analysis of fenbuconazole and its chiral metabolites in fruits, vegetables, cereals, and soil. Food Chem..

[31] Vinod Kumar V., Sonika S., Ankit M., Dipak Kumar D., Rajasekhar V.S.R.P., Renu B., Suman Y. (2024). Stereoselective analysis of chiral pesticides: A review. Environ. Monit. Assess..

[32] Dong F., Cheng L., Liu X., Xu J., Li J., Li Y., Kong Z., Jian Q., Zheng Y. (2012). Enantioselective analysis of triazole fungicide myclobutanil in cucumber and soil under different application modes by chiral liquid chromatography/tandem mass spectrometry. J. Agric. Food Chem..

[33] Jiang D., Dong F., Xu J., Liu X., Wu X., Pan X., Tao Y., Li R., Zheng Y. (2019). Enantioselective separation and dissipation of prothioconazole and its major metabolite prothioconazole-desthio enantiomers in tomato, cucumber, and pepper. J. Agric. Food Chem..

[34] Li J., Dong C., An W., Zhang Y., Zhao Q., Li Z., Jiao B. (2020). Simultaneous enantioselective determination of two new isopropanol-triazole fungicides in plant-origin foods using multiwalled carbon nanotubes in reversed-dispersive solid-phase extraction and ultrahigh-performance liquid chromatography–tandem mass spectrometry. J. Agric. Food Chem..

[35] Wang P., Jiang S., Liu D., Zhang H., Zhou Z. (2006). Enantiomeric resolution of chiral pesticides by high-performance liquid chromatography. J. Agric. Food Chem..

[36] Zhang Z., Zhang Q., Gao B., Gou G., Li L., Shi H., Wang M. (2017). Simultaneous enantioselective determination of the chiral fungicide Prothioconazole and its major chiral metabolite Prothioconazole-desthio in food and environmental samples by Ultraperformance liquid chromatography–tandem mass spectrometry. J. Agric. Food Chem..

[37] Ye X., Ma S., Zhang L., Zhao P., Hou X., Zhao L., Liang N. (2018). Trace enantioselective determination of triazole fungicides in honey by a sensitive and efficient method. J. Food Compos. Anal..

[38] Xu G., Jia X., Wu X., Xu J., Liu X., Pan X., Li R., Li X., Dong F. (2018). Enantioselective monitoring of chiral fungicide famoxadone enantiomers in tomato, apple, and grape by chiral liquid chromatography with tandem mass spectrometry. J. Sep. Sci..

[39] Zhang H., Wang X., Wang X., Qian M., Xu M., Xu H., Qi P., Wang Q., Zhuang S. (2014). Enantioselective determination of carboxyl acid amide fungicide mandipropamid in vegetables and fruits by chiral LC coupled with MS/MS. J. Sep. Sci..

[40] Zheng X., Ma W., Wang Q., Xu Y., Yang Y., Qin S., Jing X. (2024). Development of self-dispersion ferrofluid-based dispersive liquid–liquid microextraction for determining chiral fungicide hexaconazole in water, tea, and juice using high-performance liquid chromatography. Microchem. J..

[41] Bordbar M.M., Nguyen T.A., Arduini F., Bagheri H. (2020). A paper-based colorimetric sensor array for discrimination and simultaneous determination of organophosphate and carbamate pesticides in tap water, apple juice, and rice. Microchim. Acta.

[42] Zhang Y., Liu X., Qiu S., Zhang Q., Tang W., Liu H., Guo Y., Ma Y., Guo X., Liu Y. (2019). A flexible acetylcholinesterase-modified graphene for chiral pesticide sensor. J. Am. Chem. Soc..

[43] Li S., Wu Y., Ma X., Pang C., Wang M., Xu Z., Li B. (2024). Monitoring levamisole in food and the environment with high selectivity using an electrochemical chiral sensor comprising an MOF and molecularly imprinted polymer. Food Chem..

[44] Qin S., You X., Guo X., Chu H., Dong Q., Cui H., Jin F., Gao L. (2023). A chiral fluorescent COF prepared by post-synthesis modification for optosensing of imazamox enantiomers. Spectrochim. Acta Part A Mol. Biomol. Spectrosc..

[45] Yang J., Chen S.W., Zhang B., Tu Q., Wang J., Yuan M.S. (2022). Non-biological fluorescent chemosensors for pesticides detection. Talanta.

[46] Chen H., Hu O., Fan Y., Xu L., Zhang L., Lan W., Hu Y., Xie X., Ma L., She Y. (2020). Fluorescence paper-based sensor for visual detection of carbamate pesticides in food based on CdTe quantum dot and nano ZnTPyP. Food Chem..

[47] Liu J., Ye L.Y., Mo Y.Y., Yang H. (2022). Highly sensitive fluorescent quantification of acid phosphatase activity and its inhibitor pesticide Dufulin by a functional metal–organic framework nanosensor for environment assessment and food safety. Food Chem..

[48] Wang K., Wang Y., Li Q., Liu Z., Liu S. (2022). A fluorescence and localized surface plasmon resonance dual-readout sensing strategy for detection of acetamiprid and organophosphorus pesticides. Sens. Actuators B Chem..

[49] Stefan-van Staden R.I., Comnea-Stancu I.R. (2020). Chiral single-walled carbon nanotubes as chiral selectors in multimode enantioselective sensors. Chirality.

[50] Wu X., Han X., Xu Q., Liu Y., Yuan C., Yang S., Liu Y., Jiang J., Cui Y. (2019). Chiral BINOL-Based Covalent Organic Frameworks for Enantioselective Sensing. J. Am. Chem. Soc..

